# Age- and sex-related differences in risk factors for perioperative intra-aortic balloon pump application in patients undergoing coronary artery bypass grafting

**DOI:** 10.3389/fsurg.2024.1395518

**Published:** 2024-09-03

**Authors:** Junyi Gao, Qing Zhao, Yi Cheng

**Affiliations:** ^1^ Department of Cardiovascular Medicine, Beijing Shijitan Hospital Affiliated to Capital University of Medical Sciences, Beijing, China; ^2^ Department of Diagnostic Ultrasound, Beijing Anzhen Hospital Affiliated to Capital University of Medical Sciences, Beijing, China

**Keywords:** coronary artery bypass grafting (CABG), intra-aortic balloon pump (IABP), left ventricular ejection fraction (LVEF), age, sex

## Abstract

**Background:**

An intra-aortic balloon pump (IABP) is a mechanical circulatory device frequently used in patients undergoing coronary artery bypass grafting (CABG). As a treatment for perioperative haemodynamic instability, IABP insertion often implicates an adverse outcome. This study aimed to investigate the age- and sex-related disparity in risk factors for perioperative IABP insertion in CABG patients.

**Methods:**

A total of 2,460 CABG patients were included and divided into subgroups by age (elderly subgroup, ≥65 years; young subgroup, <65 years) and sex. Basic characteristics were compared between IABP and non-IABP patients in the overall patient group and the subgroups. Multivariate logistic analysis was used to investigate the significant risk factors for perioperative IABP application, and interaction effects among the potential risk factors were analysed. Combined receiver operating characteristic analysis was used to evaluate the prediction value of combined risk factors.

**Results:**

The overall patient group had a mean age of 61.5 years. The application rate of perioperative IABP was 8.0%. A left ventricular ejection fraction (LVEF) <50% significantly correlated with perioperative IABP application in the overall patient group and the subgroups. Traditional factors such as myocardial infarction history, atrial fibrillation history, and intraoperative estimated blood loss were significant risk factors in certain subgroups. Small dense low-density lipoprotein levels were significantly associated with IABP insertion in the male subgroup and young subgroup. The area under the curve of combined risk factors was significantly higher than that of LVEF <50% alone in the overall patient group and subgroups.

**Conclusion:**

Age- and sex-related differences were present in the risk factor distribution for perioperative IABP insertion in CABG patients.

## Background

Coronary artery bypass grafting (CABG) is one of the most important treatments for severe coronary atherosclerotic heart disease (1). An intra-aortic balloon pump (IABP) is a frequently used mechanical circulatory device in patients undergoing CABG (2). As an adjunctive treatment for haemodynamic instability, intra- or postoperative IABP insertion often implicates an adverse outcome of CABG (3). For developing counties, the use of an IABP brings an extra economic burden to patients.

Impaired left ventricle systolic function is a widely recognised risk factor in IABP insertion and it can be revealed by preoperative routine echocardiography (4). Other potential risk factors, such as comorbidities and specific biomarkers that have emerged recently as prognosis predictors of CABG, may also provide additive value (5). There are no well-defined criteria for perioperative IABP application, and thus it is important to identify potential controllable risk factors and improve preoperative management (6).

Recently, scholars highlighted the importance of age- and sex-related disparities in the risk factor distribution and outcomes in CABG patients (7). It is believed that patients of an advanced age may require more mechanical support during cardiac surgery, and females often had worse outcomes (8, 9). As far as we know, few studies have been conducted on age- and sex-related differences in the risk factor distribution for perioperative IABP application. This study aimed to investigate the risk factors for perioperative IABP insertion in different subgroups of CABG patients.

## Patients and methods

### Patients

**Figure 1:**
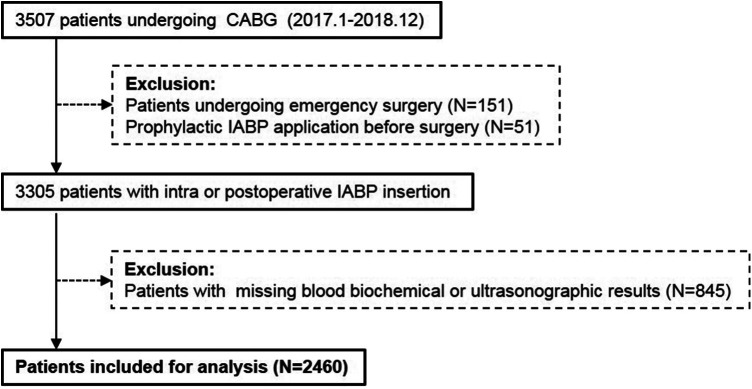
Flow chart of the study. CABG, coronary artery bypass grafting; IABP, intra-aortic balloon pump.

We retrospectively reviewed 3,507 patients (>18 years of age) undergoing CABG in the Department of Cardiac Surgery at Anzhen Hospital between 1 January 2017 and 31 December 2018. Patients undergoing emergency surgery (*N* = 151) were excluded due to incomplete preoperative data. Patients who received prophylactic preoperative IABP support were also excluded (*N* = 51). A total of 845 patients were excluded for missing blood biochemical or ultrasonographic results. Overall, 2,460 patients were included for analysis (shown in Figure 1). The overall patient group was divided into subgroups by age (elderly, ≥65 years, *N* = 988; young <65 years, *N* = 1,472) and sex (male, *N* = 1,877; female, *N* = 583). Each subgroup was subsequently divided into non-IABP and IABP groups.

Routine preoperative examinations were performed, including medical history collection, a physical examination, blood biochemical tests, an electrocardiogram, a carotid ultrasound, and echocardiography. Carotid artery stenosis (CAS) was defined as ≥50% diameter stenosis of the internal or common carotid artery according to the Society of Radiologists through ultrasound consensus (10). A left ventricular ejection fraction (LVEF) <50% was defined as a lower than normal left ventricular systolic function (11). All images were acquired by certified experienced clinicians using Philips (Bothell, WA, USA), GE (Waukesha, WI, USA), or Hitachi (Tokyo, Japan) ultrasound imaging systems. Intraoperative estimated blood loss (EBL) ≥1,000 ml (75th percentile of the overall patient group) was defined as a large amount of EBL. Patients with IABP insertion received IABP support intra or postoperatively under the following circumstances of haemodynamic instability: (1) sudden ventricular fibrillation during an operation or in an intensive care unit (ICU) that could not be corrected using medications; (2) other kinds of arrhythmia that occurred repeatedly and could not be corrected by medications; (3) a sudden decrease in blood pressure during surgery that could not be maintained using vasopressors; and (4) other signs of decreased cardiac function, including a decrease in urine output or cold feet. In-hospital all-cause death was defined as death from any cause during the period of hospitalisation.

### Statistical analysis

Continuous variables were described as mean ± standard deviation or median with interquartile range. Categorical variables were described as numbers and percentages. Univariate comparisons between groups were performed using the chi-square test for categorical variables and Student's *t*-test or a Mann–Whitney rank-sum test for continuous variables, as appropriate. Multivariate logistic regression analysis was applied to explore significant risk factors for perioperative IABP application in the overall patient group and subgroups. Potential covariates were included in adjusted models according to univariate logistic regression analysis results. A combined receiver operating characteristic (ROC) analysis was performed to analyse the association between combined risk factors and IABP application. Analyses were performed using SPSS 26.0. (SPSS, Chicago, IL, USA), and *p* < 0.05 was considered statistically significant.

## Results

### Baseline characteristics

The study included 2,460 patients (mean age of 61.5 years), the majority of which were male (76.3%). The elderly subgroup (age ≥65 years) had higher prevalences of atrial fibrillation (AF), hypertension (HTN), chronic kidney disease (CKD), and CAS. The young subgroup (age <65 years) had a higher body mass index (BMI), higher prevalence of smoking and myocardial infarction (MI) history, and higher blood lipid levels. The male subgroup had a higher prevalence of MI history and a high BMI, whereas the female subgroup had higher prevalences of diabetes mellitus (DM), HTN, and CKD and higher blood lipid levels. IABP application rates were comparable between the male and female subgroups (8.5% vs. 6.5%, *p *= 0.129) and between the elderly and young subgroups (8.8% vs. 7.5%, *p *= 0.232). The basic characteristics are summarised in Table 1.

**Table 1:** The baseline characteristics of the patients.

	Overall population *N* = 2,460	Elderly subgroup *N* = 988	Young subgroup *N* = 1,472	*p*-value	Male subgroup *N* = 1,877	Female subgroup *N* = 583	*p*-value
Age (years)	61.5 ± 8.7	69.7 ± 4.0	56.0 ± 6.4	<0.001	60.7 ± 8.9	64.3 ± 7.6	<0.001
Male (%)	76.3	68.9	81.3	<0.001	—	—	
BMI (kg/m^2^)	25.5 ± 3.1	25.1 ± 3.1	25.8 ± 3.1	<0.001	25.6 ± 3.0	25.1 ± 3.5	0.004
Family history of CAD (%)	6.1	4.5	7.3	0.004	6.4%	5.3%	0.344
HTN history (%)	65.5	70.4	62.2	<0.001	62.9%	73.8%	<0.001
DM history (%)	37.2	36.4	37.7	0.524	35.1%	44.1%	<0.001
AF history (%)	2.6	3.7	1.9	0.005	2.7%	2.6%	0.905
MI history (%)	29.1	23.7	32.7	<0.001	31.2%	22.3%	<0.001
Stroke or TIA history (%)	15.4	16.8	14.5	0.116	15.0%	16.8%	0.283
Previous PCI and stent (%)	12.7	10.4	14.3	0.005	13.2%	11.1%	0.192
CKD history (%)	4.0	7.3	1.8	<0.001	3.6%	5.3%	0.060
Smoking history (%)	51.5	39.9	59.2	<0.001	64.5%	9.6%	<0.001
Preoperative SBP (mmHg)	130.4 ± 16.5	131.7 ± 16.8	129.5 ± 16.2	0.001	130.1 ± 16.6	131.3 ± 16.1	0.132
Preoperative DBP (mmHg)	75.4 ± 9.7	74.6 ± 9.4	76.0 ± 9.8	0.001	75.7 ± 9.9	74.6 ± 8.9	0.015
Preoperative heart rate (beats/min)	75.5 ± 10.4	75.6 ± 10.6	75.4 ± 10.2	0.606	75.3 ± 10.2	76.0 ± 10.7	0.162
WBC (G/L)	6.87 ± 1.68	6.83 ± 1.67	6.89 ± 1.69	0.376	6.87 ± 1.68	6.86 ± 1.70	0.872
CREA (μmol/L)	75.5 ± 21.8	76.3 ± 18.2	75.1 ± 23.9	0.180	79.4 ± 22.2	63.0 ± 14.4	<0.001
eGFR(ml/min/1.73 m^2^)	89.7 ± 14.9	82.7 ± 13.9	94.4 ± 13.7	<0.001	90.5 ± 14.9	87.1 ± 14.9	<0.001
UA (μmol/L)	329.6 ± 86.6	322.3 ± 83.9	334.4 ± 88.1	0.001	341.5 ± 84.4	291.3 ± 82.4	<0.001
TGs (mmol/L)	1.39 (1.02–1.98)	1.29 (0.95–1.77)	1.49 (1.09–2.11)	<0.001	1.37 (0.99–1.95)	1.46 (1.12–2.06)	0.203
TCHO (mmol/L)	4.15 ± 1.11	4.03 ± 1.00	4.24 ± 1.17	<0.001	4.05 ± 1.07	4.47 ± 1.18	<0.001
HDL-C (mmol/L)	1.00 ± 0.24	1.03 ± 0.24	0.97 ± 0.23	<0.001	0.98 ± 0.22	1.07 ± 0.25	<0.001
LDL-C (mmol/L)	2.52 ± 0.93	2.41 ± 0.82	2.60 ± 0.98	<0.001	2.48 ± 0.90	2.68 ± 1.00	<0.001
Sd-LDL (mmol/L)	0.73 ± 0.33	0.69 ± 0.31	0.75 ± 0.34	<0.001	0.73 ± 0.32	0.73 ± 0.36	0.660
hs-CRP (mg/L)	1.60 (0.65–4.29)	1.48 (0.62–4.25)	1.69 (0.66–4.37)	0.373	1.59 (0.64–4.25)	1.64 (0.66–4.67)	0.227
Preoperative LVEF (%)	59.9 ± 9.5	60.9 ± 8.9	59.3 ± 9.8	<0.001	59.3 ± 9.7	62.0 ± 8.6	<0.001
LVEF <50% (%)	13.0	10.5	14.8	0.002	14.3	9.0	0.001
CAS (%)	28.5	35.6	23.7	<0.001	28.9	27.3	0.454
EuroSCORE	3 (1–4)	4 (3–6)	2 (1–3)	<0.001	2 (1–4)	4 (2–5)	<0.001
Off-pump CABG (%)	10.4	11.1	10.0	0.362	10.6	9.9	0.652
Duration of surgery (h)	4.29 ± 0.99	4.29 ± 1.04	4.29 ± 0.96	0.938	4.29 ± 1.00	4.28 ± 0.97	0.834
Intraoperative EBL	760 (600–1,000)	760 (600–1,000)	800 (600–1,000)	0.272	800 (600–1,000)	700 (600–900)	0.026
intraoperative EBL ≥1,000 ml	27.2	26.7	27.5	0.665	28.5	23.0	0.009
Length of ICU stay (h)	21 (17–27)	22 (18–30)	21 (17–26)	0.004	21 (17–26)	22 (18–29)	0.122
Length of hospital stay (days)	15 (12–19)	15 (12–20)	15 (12–19)	0.243	15 (12–19)	16 (12–20)	0.213
IABP application (%)	8.0	8.8	7.5	0.232	8.5	6.5	0.129
In-hospital all-cause deaths (%)	1.6	2.4	1.1	0.010	1.5	1.9	0.569

BMI, body mass index; CAD, coronary artery disease; HTN, hypertension; DM, diabetes mellitus; AF, atrial fibrillation; MI, myocardial infarction; TIA, transient ischaemic attack; PCI, percutaneous coronary intervention; CKD, chronic kidney disease; SBP, systolic blood pressure; DBP, diastolic blood pressure; WBC, white blood cell; CREA, creatinine; eGFR, estimated glomerular filtration rate; UA, uric acid; TGs, triglycerides; TCHO, total cholesterol; HDL-C, high density lipoprotein cholesterol; LDL-C, low-density lipoprotein cholesterol; sd-LDL, small dense low-density lipoprotein; hs-CRP, high sensitive c-reactive protein; LVEF, left ventricular ejection fraction; CAS, carotid artery stenosis; EuroSCORE, European System for Cardiac Operative Risk Evaluation; CABG, coronary artery bypass grafting; EBL, estimated blood loss; IABP, intra-aortic balloon pump.

### Comparison between IABP and non-IABP patients

In the overall patient group, IABP patients had higher prevalences of HTN, AF, and MI history, higher baseline uric acid (UA) and small dense low-density lipoprotein (sd-LDL) levels, and lower baseline systolic blood pressure (SBP), BMI, and LVEF. In the elderly subgroup, there were a higher proportion of males in the IABP patients and higher prevalences of AF and MI history. In the young subgroup, IABP patients had a higher prevalence of MI history, higher baseline UA and sd-LDL levels, and lower BMI. In the male subgroup, IABP patients had higher prevalences of AF and MI history, and higher baseline UA and sd-LDL levels. In the female subgroup, IABP patients had age, comorbidities, and blood lipid levels that were comparable with non-IABP patients, although they had a lower baseline LVEF.

In addition, in the overall patient group and subgroups, IABP patients generally had a lower baseline LVEF, a larger amount of intraoperative EBL, longer hospital and ICU stays, and a higher incidence of in-hospital all-cause death. A comparison of the baseline characteristics between IABP and non-IABP patients is summarised in Table 2.

**Table 2:** Comparison of the characteristics of IABP and non-IABP patients in subgroups.

	Overall population			Elderly subgroup			Young subgroup			Male subgroup			Female subgroup		
	Non-IABP *N* = 2,263	IABP *N* = 197	*p*-value	Non-IABP *N* = 901	IABP *N* = 87	*p*-value	Non-IABP *N* = 1,362	IABP *N* = 110	*p*-value	Non-IABP *N* = 1,718	IABP *N* = 159	*p*-value	Non-IABP *N* = 545	IABP *N* = 38	*p*-value
Age (years)	61.5 ± 8.7	61.6 ± 9.1	0.845	69.7 ± 3.9	69.6 ± 4.7	0.851	56.1 ± 6.4	55.3 ± 6.4	0.223	60.6 ± 8.8	61.1 ± 9.5	0.488	64.3 ± 7.6	63.7 ± 7.3	0.662
Male (%)	75.9	80.7	0.129	67.9	79.3	0.028	81.2	81.8	0.874	—	—	—	—	—	—
BMI (kg/m^2^)	25.5 ± 3.1	25.1 ± 3.0	0.042	25.1 ± 3.1	25.1 ± 3.3	0.863	25.8 ± 3.1	25.1 ± 2.7	0.015	25.7 ± 2.9	25.1 ± 3.0	0.015	25.1 ± 3.5	25.1 ± 3.1	0.917
Family history of CAD (%)	6.3	4.1	0.205	4.6	3.4	0.838	7.5	4.5	0.253	6.6	3.8	0.158	5.3	5.3	0.988
HTN history (%)	66.0	59.4	0.061	70.7	67.8	0.574	62.9	52.7	0.034	63.6	56.0	0.058	73.8	73.7	0.992
DM history (%)	37.6	32.5	0.154	37.1	29.9	0.184	38.0	34.5	0.477	35.4	30.8	0.242	44.4	39.5	0.554
AF history (%)	2.5	4.6	0.079	3.2	9.2	0.012	2.0	0.9	0.667	2.4	5.0	0.093	2.6	2.6	0.981
MI history (%)	27.9	42.1	<0.001	22.1	40.2	<0.001	31.8	43.6	0.011	29.9	45.3	<0.001	21.8	28.9	0.308
Stroke or TIA history (%)	15.6	13.2	0.371	16.6	18.4	0.678	14.9	9.1	0.127	15.2	12.6	0.377	16.9	15.8	0.862
Previous PCI (%)	12.4	16.2	0.122	10.2	12.6	0.478	13.9	19.1	0.133	12.8	17.6	0.087	11.2	10.5	0.900
CKD history (%)	3.9	4.6	0.665	7.5	5.7	0.558	1.6	3.6	0.241	3.5	4.4	0.557	5.3	5.3	0.988
Smoking history (%)	51.4	51.8	0.927	40.1	37.9	0.698	59.0	62.7	0.439	64.6	62.9	0.665	9.9	5.3	0.513
Preoperative SBP (mmHg)	130.6 ± 16.5	127.9 ± 16.3	0.028	131.8 ± 17.0	130.7 ± 15.1	0.548	129.8 ± 16.1	125.7 ± 17.0	0.011	130.3 ± 16.5	127.8 ± 17.0	0.073	131.5 ± 16.3	128.2 ± 13.4	0.222
Preoperative DBP (mmHg)	75.5 ± 9.7	75.1 ± 9.7	0.575	74.6 ± 9.4	75.5 ± 9.6	0.360	76.1 ± 9.8	74.7 ± 9.8	0.159	75.7 ± 9.8	75.3 ± 9.9	0.581	74.7 ± 9.0	74.2 ± 8.5	0.758
Preoperative heart rate (beat/min)	75.6 ± 10.4	74.8 ± 9.4	0.291	75.6 ± 10.6	75.7 ± 10.3	0.960	75.5 ± 10.3	74.0 ± 8.6	0.135	75.4 ± 10.3	74.9 ± 9.7	0.609	76.2 ± 10.8	74.0 ± 8.0	0.118
WBC (G/L)	6.88 ± 1.69	6.74 ± 1.66	0.283	6.84 ± 1.68	6.70 ± 1.59	0.445	6.90 ± 1.69	6.78 ± 1.71	0.465	6.87 ± 1.68	6.88 ± 1.69	0.968	6.90 ± 1.7	6.19 ± 1.4	0.012
CREA (μmol/L)	75.4 ± 22.1	77.0 ± 17.7	0.336	76.2 ± 18.5	77.1 ± 15.8	0.645	74.9 ± 24.2	76.9 ± 19.2	0.412	79.3 ± 22.7	80.3 ± 17.2	0.599	63.0 ± 14.5	63.0 ± 12.7	0.988
eGFR (ml/min/1.73 m^2^)	89.8 ± 15.0	89.4 ± 14.6	0.745	82.6 ± 14.0	83.9 ± 13.3	0.416	94.5 ± 13.7	93.8 ± 14.3	0.595	90.6 ± 14.9	89.8 ± 14.7	0.541	87.1 ± 14.9	87.5 ± 14.6	0.868
UA (μmol/L)	328.5 ± 86.4	342.0 ± 88.8	0.036	321.3 ± 84.6	333.1 ± 75.4	0.210	333.2 ± 87.2	349.0 ± 97.9	0.072	340.2 ± 84.7	355.2 ± 81.0	0.032	291.6 ± 81.2	286.5 ± 99.4	0.711
TGs (mmol/L)	1.40 (1.03–2.00)	1.36 (0.96–1.83)	0.009	1.30 (0.96–1.76)	1.26 (0.89–1.78)	0.969	1.50 (1.10–2.15)	1.47 (1.03–1.89)	0.023	1.37 (1.00–1.97)	1.38 (0.91–1.83)	0.126	1.47 (1.14–2.07)	1.24 (1.01–1.84)	0.120
TCHO (mmol/L)	4.16 ± 1.11	4.10 ± 1.12	0.511	4.04 ± 1.00	3.89 ± 0.92	0.186	4.23 ± 1.16	4.27 ± 1.23	0.760	4.06 ± 1.06	4.01 ± 1.10	0.615	4.47 ± 1.18	4.47 ± 1.14	0.972
HDL-C (mmol/L)	1.00 ± 0.23	1.01 ± 0.24	0.575	1.04 ± 0.25	1.00 ± 0.23	0.205	0.97 ± 0.22	1.01 ± 0.25	0.074	0.98 ± 0.23	0.98 ± 0.22	0.975	1.06 ± 0.25	1.13 ± 0.31	0.102
LDL-C (mmol/L)	2.52 ± 0.93	2.55 ± 0.91	0.656	2.42 ± 0.83	2.34 ± 0.73	0.404	2.59 ± 0.98	2.72 ± 1.01	0.184	2.47 ± 0.90	2.51 ± 0.90	0.569	2.68 ± 1.00	2.71 ± 0.99	0.845
sd-LDL (mmol/L)	0.72 ± 0.33	0.79 ± 0.35	0.005	0.69 ± 0.30	0.73 ± 0.35	0.195	0.75 ± 0.34	0.84 ± 0.34	0.006	0.72 ± 0.32	0.78 ± 0.33	0.017	0.73 ± 0.36	0.82 ± 0.42	0.123
Hs-CRP (mg/L)	1.60 (0.64–4.28)	1.55 (0.78–4.70)	0.380	1.48 (0.61–4.23)	1.47 (0.69–4.88)	0.474	1.69 (0.65–4.35)	1.78 (0.83–4.65)	0.566	1.59 (0.63–4.25)	1.54 (0.77–4.35)	0.686	1.64 (0.65–4.56)	1.73 (0.78–6.65)	0.233
Preoperative LVEF (%)	60.5 ± 8.9	53.3 ± 12.8	<0.001	61.4 ± 8.5	56.3 ± 11.6	<0.001	59.9 ± 9.2	50.9 ± 13.3	<0.001	59.9 ± 9.0	52.0 ± 13.0	<0.001	62.3 ± 8.4	58.9 ± 10.4	0.057
LVEF <50% (%)	11.0	36.0	<0.001	9.2	24.1	<0.001	12.3	45.5	<0.001	12.0	39.0	<0.001	8.0	23.7	0.001
CAS (%)	28.1	32.5	0.196	35.5	36.8	0.814	23.3	29.1	0.168	28.3	35.2	0.065	27.7	21.1	0.373
EuroSCORE	3 (1–4)	3 (2–5)	<0.001	4 (3–5)	5 (3–6)	0.013	2 (1–3)	3 (1–4)	<0.001	2 (1–4)	3 (2–5)	<0.001	4 (2–5)	4 (2–5)	0.713
Off-pump CABG (%)	10.5	10.2	0.888	11.3	9.2	0.547	9.9	10.9	0.737	10.5	11.3	0.758	10.3	5.3	0.318
Intraoperative EBL	700 (600–1,000)	800 (600–1,000)	0.009	700 (600–1,000)	800 (600–1,000)	0.009	700 (600–1,000)	800 (600–1,000)	0.050	800 (600–1,000)	800 (600–1,000)	0.007	700 (600–900)	800 (600–1,000)	0.127
Intraoperative EBL ≥1,000 ml	26.3	37.1	0.001	25.6	37.9	0.013	26.8	36.4	0.031	27.6	38.4	0.004	22.4	31.6	0.193
Duration of surgery (h)	4.27 ± 0.96	4.52 ± 1.27	0.009	4.27 ± 1.03	4.55 ± 1.10	0.015	4.3	4.5	0.119	4.28	4.45	0.113	4.25	4.80	0.001
Length of ICU stay (h)	21 (17–25)	50 (25–96)	<0.001	21 (17–26)	51 (25–110)	<0.001	21 (17–25)	50 (25–90)	<0.001	21 (17–25)	50 (25–96)	<0.001	21 (18–26)	49 (24.5–108.5)	<0.001
Length of hospital stay (days)	15 (12–19)	18 (14–23)	<0.001	15 (12–19)	17 (15–23)	<0.001	15 (12–19)	19 (14.24)	<0.001	15 (12–19)	18 (14–24)	<0.001	16 (12–20)	17 (13.5–21.5)	0.162
In-hospital all-cause deaths (%)	0.7	12.2	<0.001	1.1	16.1	<0.001	0.4	9.1	<0.001	0.6	11.9	<0.001	1.1	13.2	<0.001

BMI, body mass index; CAD, coronary artery disease; HTN, hypertension; DM, diabetes mellitus; AF, atrial fibrillation; MI, myocardial infarction; TIA, transient ischaemic attack; PCI, percutaneous coronary intervention; CKD, chronic kidney disease; SBP, systolic blood pressure; DBP, diastolic blood pressure; WBC, white blood cell; CREA, creatinine; eGFR, estimated glomerular filtration rate; UA, uric acid; TGs, triglycerides; TCHO, total cholesterol; HDL-C, high density lipoprotein cholesterol; LDL-C, low-density lipoprotein cholesterol; Sd-LDL, small dense low-density lipoprotein; hs-CRP, high sensitive c-reactive protein; LVEF, left ventricular ejection fraction; CAS, carotid artery stenosis; EuroSCORE, European System for Cardiac Operative Risk Evaluation; CABG, coronary artery bypass grafting; EBL, estimated blood loss; IABP, intra-aortic balloon pump.

### Risk factors for perioperative IABP application in the overall patient group

**Figure 2:**
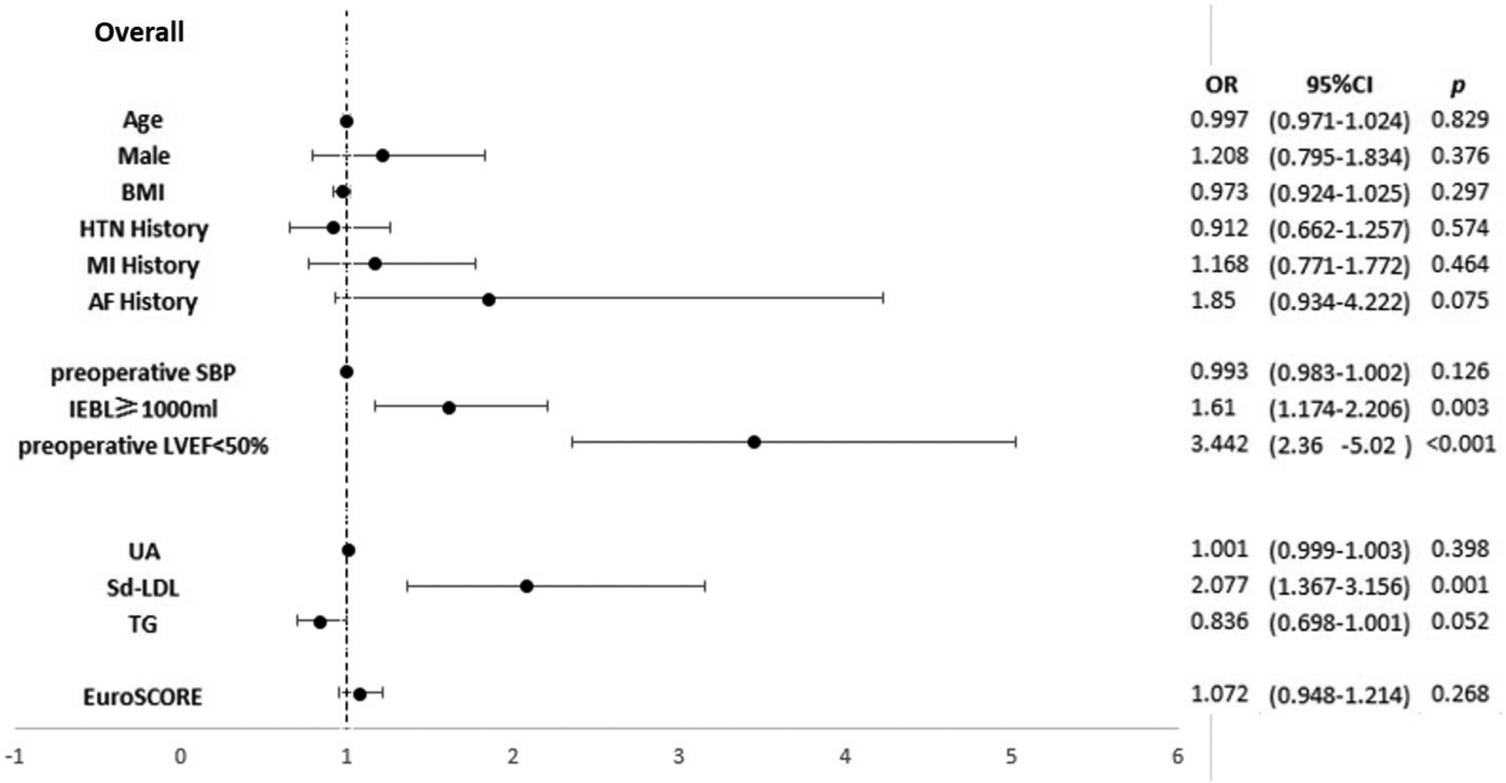
Forest plot of a multivariate logistic regression analysis of risk factors associated with perioperative IABP insertion in the overall patient group. LVEF <50% (OR, 3.44; *p *< 0.001), sd-LDL levels (OR, 2.08; *p *= 0.001), and EBL ≥1,000 ml (OR, 1.61; *p *= 0.003) were significant risk factors.

In the overall patient group, univariate logistic analysis showed that BMI, HTN history, MI history, AF history, preoperative SBP, UA, sd-LDL, triglycerides (TGs), LVEF <50%, EBL ≥1,000 ml, and European System for Cardiac Operative Risk Evaluation (EuroSCORE) significantly correlated with the application of a perioperative IABP. Multivariate logistic analysis showed that LVEF <50% (OR, 3.44; *p *< 0.001), sd-LDL (OR, 2.08; *p *= 0.001), and EBL ≥1,000 ml (OR, 1.61; *p *= 0.003) were significant risk factors. LVEF <50% and sd-LDL had a synergistic effect (OR, 5.71; *p *< 0.001). Results are shown in Supplementary Tables S1, S2 and Figure 2.

### Differences in risk factors for perioperative IABP application in subgroups

**Figure 3:**
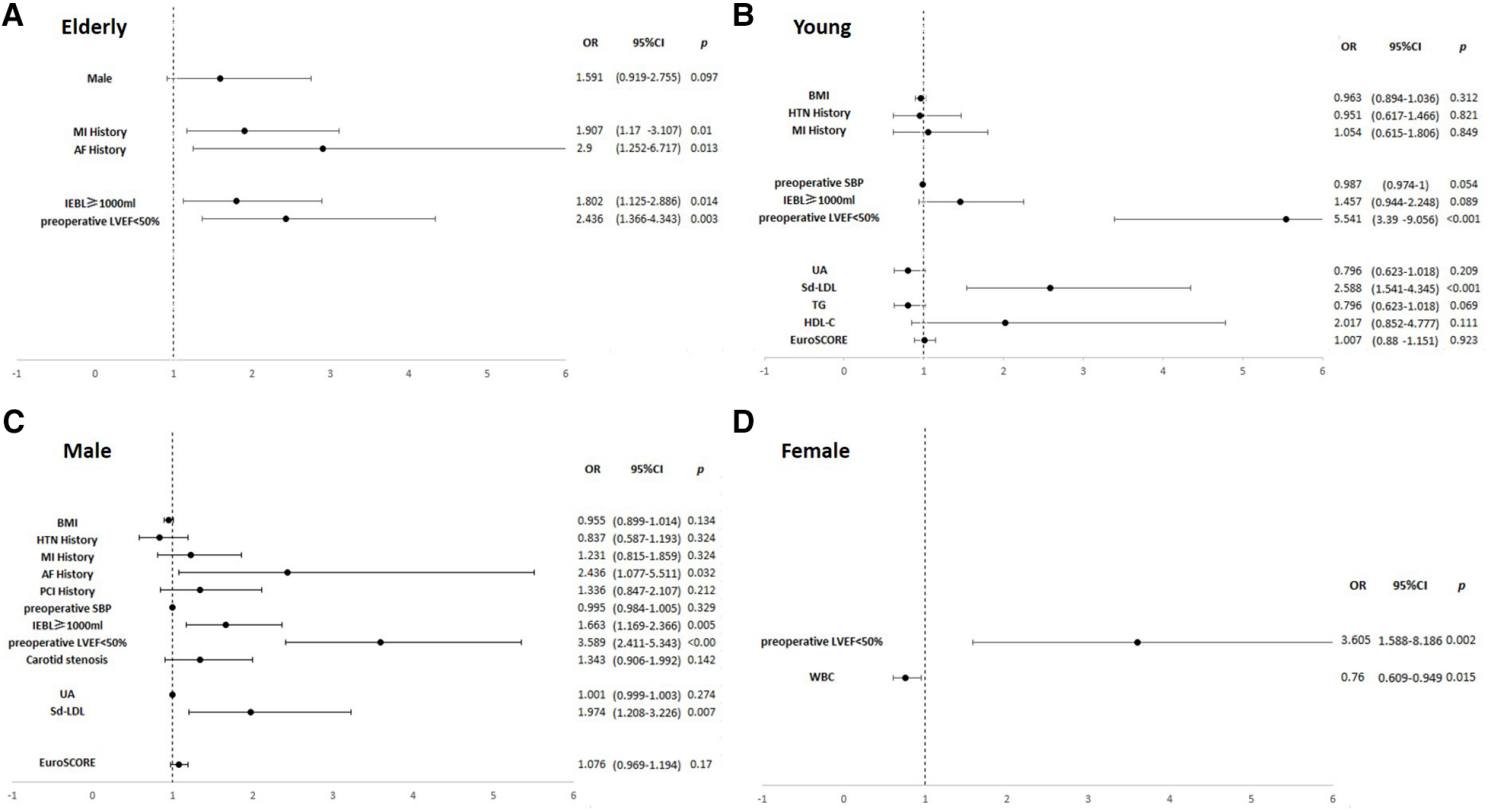
Forest plots of multivariate logistic regression analyses of risk factors associated with perioperative IABP insertion in the subgroups. **(A)** The elderly subgroup. **(B)** The young subgroup. **(C)** The male subgroup. **(D)** The female subgroup. In the elderly subgroup, MI history (OR, 1.91; *p *= 0.010), AF history (OR, 2.90; *p *= 0.013), LVEF <50% (OR, 2.44; *p *= 0.003), and EBL ≥1,000 ml (OR, 1.80; *p *= 0.014) were significant risk factors. In the young subgroup, sd-LDL (OR, 2.59; *p *< 0.001) and LVEF <50% (OR, 5.54; *p *< 0.001) were significant risk factors. In the male subgroup, AF history (OR, 2.44; *p *= 0.032), sd-LDL levels (OR, 1.97; *p *= 0.007), LVEF <50% (OR, 3.59; *p *< 0.001), and EBL ≥1,000 ml (OR, 1.66; *p *= 0.005) were significant risk factors. In the female subgroup, WBC (OR, 0.760; *p *= 0.015) and LVEF <50% (OR, 3.61; *p *= 0.002) were significant risk factors.

In the elderly subgroup, MI history (OR, 1.91; *p *= 0.010), AF history (OR, 2.90; *p *= 0.013), LVEF <50% (OR, 2.44; *p *= 0.003), and EBL ≥1,000 ml (OR, 1.80; *p *= 0.014) were significant risk factors for perioperative IABP application. LVEF <50% had a synergistic effect with AF history (OR, 6.99, *p *= 0.034) and MI history (OR, 4.36, *p *< 0.001). In the young subgroup, sd-LDL (OR, 2.59; *p *< 0.001) emerged as a significant risk factor along with LVEF <50% (OR, 5.54; *p *< 0.001). LVEF <50% and sd-LDL had a synergistic effect (OR, 7.66; *p *< 0.001). In the male subgroup, AF history (OR, 2.44; *p *= 0.032), sd-LDL levels (OR, 1.97; *p *= 0.007), LVEF <50% (OR, 3.59; *p *< 0.001), and EBL ≥1,000 ml (OR, 1.66; *p *= 0.005) were significant risk factors. LVEF <50% and sd-LDL had a synergistic effect (OR, 5.57; *p *< 0.001). In the female subgroup, white blood cell (WBC) (OR, 0.760; *p *= 0.015) and LVEF <50% (OR, 3.61; *p *= 0.002) were significant risk factors. Results are shown in Supplementary Tables S1, S2 and Figure 3.

### ROC analyses

**Figure 4:**
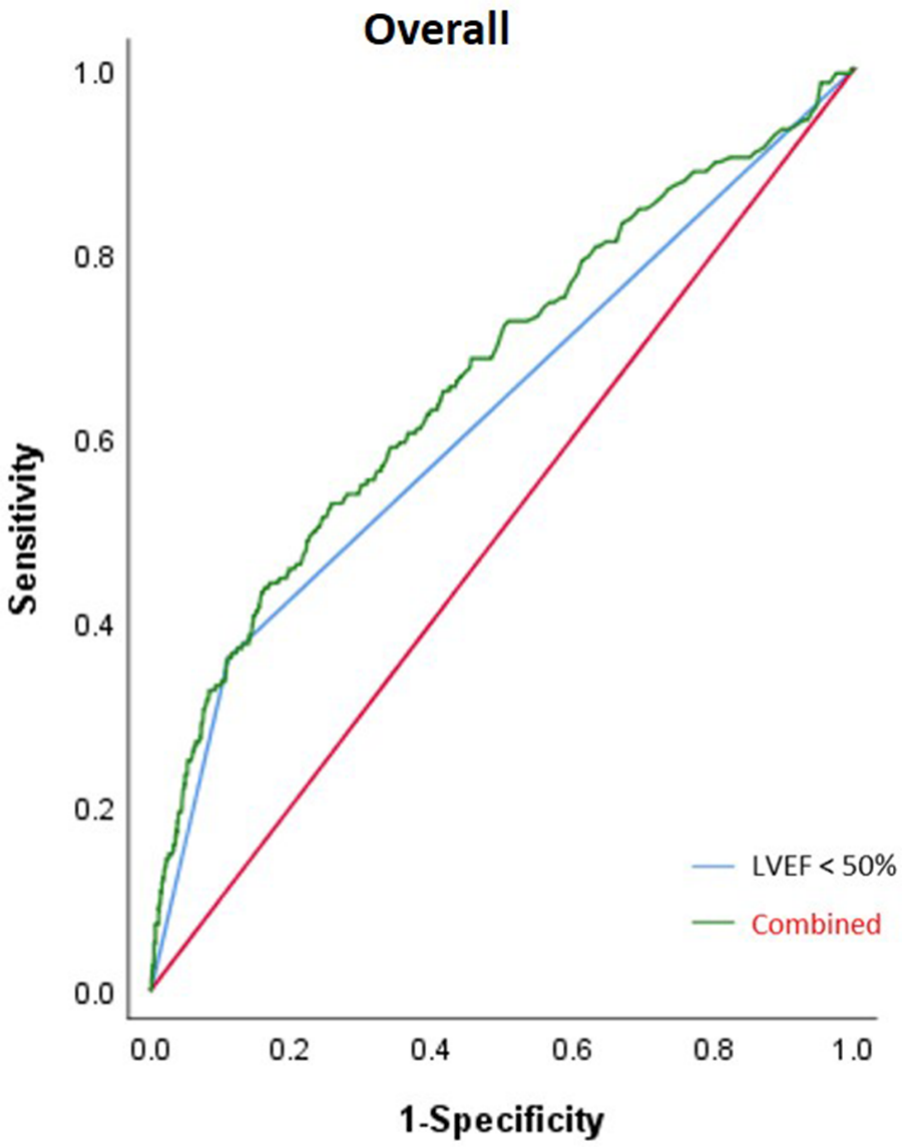
Result from the ROC analysis of the overall patient group. The AUC of LVEF <50% for IABP application was 0.625 (*p *< 0.001) and the combined AUC of LVEF <50%, intraoperative EBL ≥1,000 ml, and sd-LDL was 0.669 (*p *< 0.001).

**Figure 5:**
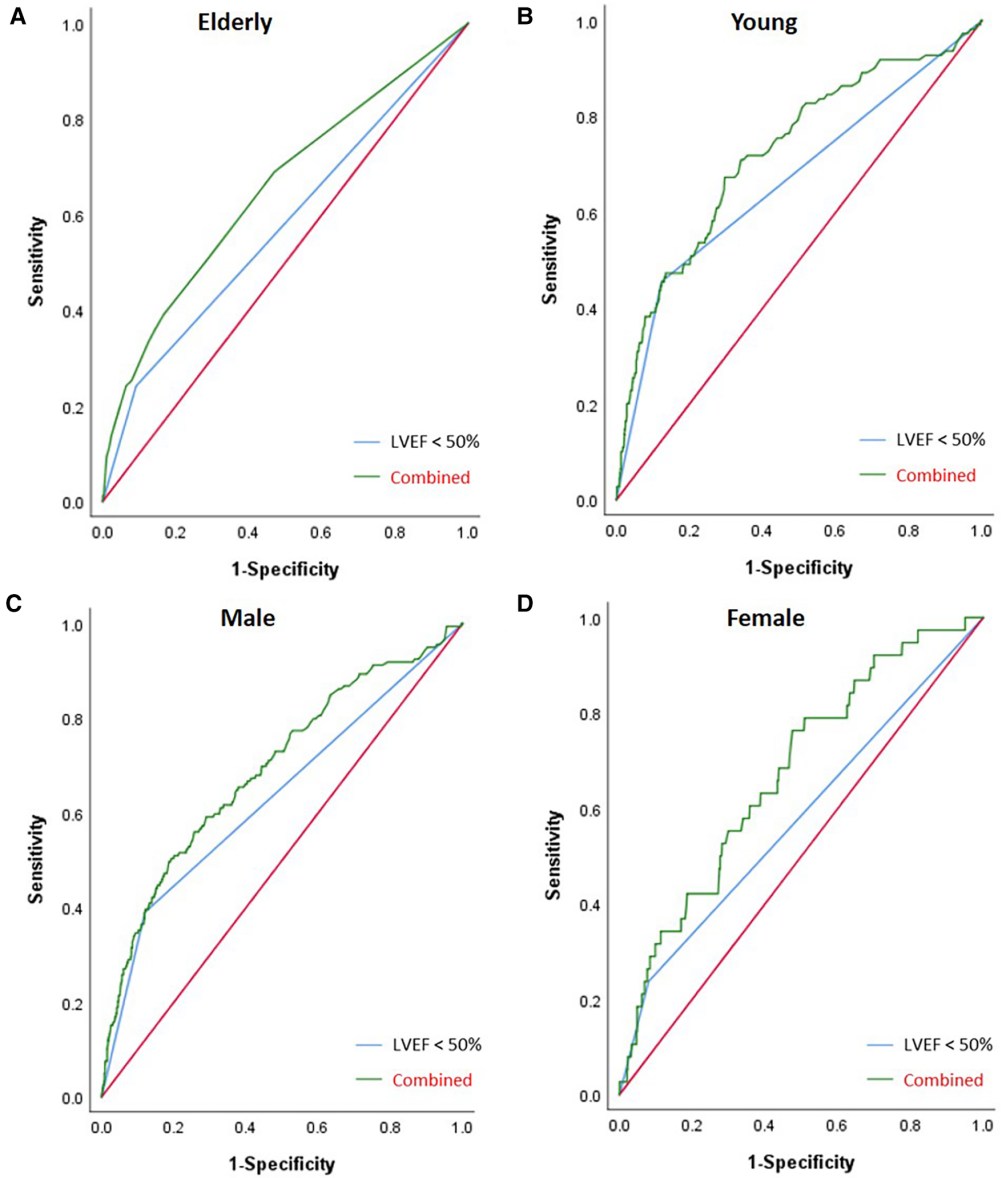
Results from ROC analyses of the subgroups. **(A)** The elderly subgroup. **(B)** The young subgroup. **(C)** The male subgroup. **(D)** The female subgroup. In the elderly subgroup, the AUC of LVEF <50% was 0.575 (*p *= 0.001) and the combined AUC of AF history, MI history, LVEF <50%, and intraoperative EBL ≥1,000 ml was 0.653 (*p *< 0.001). In the young subgroup, the AUC of LVEF <50% was 0.666 (*p *< 0.001) and the combined AUC of LVEF <50% and sd-LDL was 0.724 (*p *< 0.001). In the male subgroup, the AUC of LVEF <50% was 0.635 (*p *= 0.020) and the combined AUC of AF history, LVEF <50%, sd-LDL, and intraoperative EBL ≥1,000 ml was 0.693 (*p *= 0.024). In the female subgroup, the AUC of LVEF <50% was 0.579 (*p* = 0.027) and the combined AUC of LVEF <50% and WBC was 0.675 (*p *< 0.001). The AUC of the combined risk factors was significantly higher than that of LVEF alone in each subgroup (*p* < 0.01).

In the overall patient group, the area under the curve (AUC) of LVEF <50% for IABP application was 0.625 [95% confidence interval (CI) (0.591–0.659); *p *< 0.001] and the combined AUC of LVEF <50%, EBL ≥1,000 ml, and sd-LDL was 0.669 [95% CI (0.626–0.712); *p *< 0.001]. In the elderly subgroup, the AUC of LVEF <50% was 0.575 [95% CI (0.529–0.621); *p *= 0.001] and the combined AUC of AF history, MI history, LVEF <50%, and EBL ≥1,000 ml was 0.653 [95% CI (0.591–0.715); *p *< 0.001]. In the young subgroup, the AUC of LVEF <50% was 0.666 [95% CI (0.618–0.714); *p *< 0.001] and the combined AUC of LVEF <50% and sd-LDL was 0.724 [95% CI (0.671–0.778); *p *< 0.001]. In the male subgroup, the AUC of LVEF <50% was 0.635 [95% CI (0.596–0.674); *p *= 0.020] and the combined AUC of AF history, LVEF <50%, sd-LDL, and EBL ≥1,000 ml was 0.693 [95% CI (0.646–0.739); *p *= 0.024]. In the female subgroup, the AUC of LVEF <50% was 0.579 [95% CI (0.509–0.648); *p *= 0.027] and the combined AUC of LVEF <50% and WBC was 0.0.675 [95% CI (0.588–0.762); *p *< 0.001]. The AUC of the combined risk factors was significantly higher than that of LVEF alone in each subgroup. Results of the ROC analysis are shown in Table 3 and Figures 4, 5.

**Table 3:** ROC analyses of risk factors for perioperative IABP application in the overall population and subgroups.

	Preoperative LVEF <50%				Combined risk factors				Comparison of AUC		
	AUC	*p-*value	95% CI		AUC	*p-*value	95% CI		AUC difference	*Z*-value	*p-*value
Overall population	0.625	<0.001	0.591	0.659	0.669	<0.001	0.626	0.712	0.044	2.836	0.005
Elderly subgroup	0.575	0.001	0.529	0.621	0.653	<0.001	0.591	0.715	0.078	2.998	0.003
Young subgroup	0.666	<0.001	0.618	0.714	0.724	<0.001	0.671	0.778	0.058	3.136	0.002
Male subgroup	0.635	0.020	0.596	0.674	0.693	0.024	0.646	0.739	0.058	3.479	0.001
Female subgroup	0.579	0.027	0.509	0.648	0.675	<0.001	0.588	0.762	0.096	2.632	0.008

ROC, receiver operating characteristic; LVEF, left ventricular ejection fraction; AUC, area under the curve.

## Discussion

The most important findings of our study include the following: (1) in different subgroups, different factors provided complementary values to a lower baseline LVEF, a generally accepted risk factor for perioperative IABP insertion; (2) the AUC of combined risk factors in each subgroup was significantly higher than that of LVEF alone; and (3) besides traditional risk factors, such as MI history or AF history, baseline sd-LDL level emerged as a risk factor in certain subgroups.

There were no well-defined criteria for perioperative IABP insertion and multiple factors should be taken into consideration (9). As an important haemodynamic parameter, LVEF was recognised as a predictor of a poor prognosis for CABG patients. Fallahzadeh et al. indicated that patients with a severely reduced baseline LVEF were at a higher risk of mortality after CABG (12). Recently, Kumar et al. proposed that preoperative three-dimensional LVEF could predict intra and postoperative IABP insertion in CABG patients (4). Our result showed that a lower than normal baseline LVEF (<50%) was significantly associated with perioperative IABP application in the overall patient group and subgroups, which was consistent with previous studies.

In addition to traditional risk factors, our data revealed that baseline sd-LDL significantly correlated with IABP insertion in the young subgroup and male subgroup. Krychtiuk et al. proposed that among coronary artery disease (CAD) patients with high sd-LDL levels, monocyte subset distribution is skewed to a more “pro-inflammatory” profile (13). These cells then respond upon activation with a higher production of inflammatory cytokines (14, 15). During major surgeries such as CABG, the cardiovascular system develops specific reactions (activation of the inflammatory cascade, which involves numerous cytokines and chemokines) against the stress (16, 17). The inflammatory responses induced by surgery might contribute to unstable haemodynamics when IABP is needed (18, 19). Therefore, an elevated level of sd-LDL may exert its effects through the modulation of the monocyte subset distribution to a rather pro-inflammatory profile. In addition, Norata et al. proposed that sd-LDL can induce inflammatory responses in endothelial cells, and subsequent endothelial dysfunction makes it difficult for the cardiovascular system to adapt to the haemodynamic changes after major surgeries (20).

The timing and intensity of the response mentioned above vary among individuals. For instance, patients of different ages may have different oxidative stress statuses (different cytokine and chemokine levels), which may lead to different responses to the stress (21). In addition, studies have proposed that the drivers of coronary microvascular dysfunction may differ by sex. Inflammation predominates in males, whereas ventricular remodelling and fibrosis play a major role in females (22). In our study, the role of sd-LDL was more prominent in the young subgroup and male subgroup. Our result indicated that physicians may pay more attention to the inflammatory background before cardiac surgery in certain subgroups.

Our data failed to reveal the significant correlation of sd-LDL with perioperative IABP insertion in females and the elderly subgroup. We speculate that one of the possible explanations might be that the distributions of sd-LDL were also influenced by age, sex, and menopausal status. In males, sd-LDL levels showed an increasing phase followed by a decreasing phase and the summit was reached at approximately 60 years of age. In females, the increasing phase was followed by a plateaued phase, which may have occurred due to the impact of the postmenopausal status (23). Therefore, we speculate that the decrease in sd-LDL after middle age and the impact of the menopausal status may have concealed the role of sd-LDL in elderly and female patients. In addition, female CABG patients had more comprehensive comorbidities, and the drugs they routinely took may have had a certain impact on sd-LDL levels (24).

Previous studies have reported that sd-LDL is correlated with CAD severity and a higher incidence of adverse events. One of the possible explanations might be that sd-LDL promotes atherosclerosis plaque progression through its strong pro-atherosclerotic effect (24–26). Therefore, we assume that the association of sd-LDL with IABP application may be attributed the fact that patients with a higher sd-LDL level may have more severe CAD, which leads to a subsequent IABP insertion. However, CAD severity is often estimated by angiography. In our data, all patients (CABG patients) had established severe CAD; we assume it may be not easy to stratify the severity using only imaging results. Sd-LDL level may serve as a complementary factor to help stratify the risk of CABG patients.

Finally, we found synergistic effects between certain potential risk factors. LVEF <50% and sd-LDL could promote each other's effect in the overall patient group, the male subgroup, and the younger subgroup. We assume the possible explanation may be that abnormal lipid metabolism influences cardiac function through various mechanisms. Previous studies have reported that lipotoxicity can promote stiffening and inflammation of the cardiac tissue (27). Although pathological evidence is lacking, our result revealed that patients with higher sd-LDL levels and a lower LVEF were more likely to experience haemodynamic instability. However, we have to admit that LVEF could be influenced by several factors, and the synergistic effect between LVEF and sd-LDL has to be confirmed in future studies. These two factors were not significantly associated with IABP use in the elderly subgroup, perhaps due to both being influenced by an abnormal lipid metabolism; young patients may have more obvious consequences as the cardiac function of the elderly has already been impaired by other factors, such as ageing. Therefore, physicians should pay some attention to lipid metabolism in young and male patients in addition to their cardiac function and comorbidity. In the elderly group, we found MI history and AF history both had a synergistic effect with lower LVEF. This result was consistent with clinical understanding. However, our study only included in-hospital mortality, and as for long-term mortality, further study is needed. For instance, in this CABG population, there were patients with preserved LVEF, such as those with heart failure with a preserved ejection fraction (HFpEF), who may have even worse long-term outcomes (28).

The study had some limitations. First, our study was a single-centre study that indicated the situation in one hospital. Therefore, we only provided a preliminary result of IABP risk prediction in CABG patients, which needs to be validated in a larger population. Second, we excluded patients with preoperative IABP insertion. As there is no well-defined guideline, prophylactic IABP may involve the physician’s personal judgement. The third limitation was that we used EuroSCORE instead of EuroSCORE II due to incomplete information. In our study, we aimed to reveal the preoperative risk of the patients and we assume EuroSCORE may be sufficient. The next limitation was that the influence of drugs was not taken into consideration. A large proportion of patients took certain drugs, such as antidiabetic or antihyperlipidemic drugs, which may affect their baseline levels of biochemical markers. However, the effect was not easy to evaluate.

## Conclusion

Our study confirmed the significant correlation of traditional risk factors (LVEF, MI history, AF history, and intraoperative EBL) with perioperative IABP insertion, and baseline sd-LDL level emerged as a biochemical risk factor in certain subgroups. In addition, we found a disparity in risk factors; different factors provided complementary values to a lower baseline LVEF in different subgroups. Our result provided complementary knowledge to this field, and it is crucial to be aware of the difference in risk factor distribution in subgroups during the preoperative evaluation of CABG patients.

## Data Availability

The raw data supporting the conclusions of this article will be made available by the authors, without undue reservation.
