# Supplementary material for: Age- and sex-related differences in risk factors for perioperative intra-aortic balloon pump application in patients undergoing coronary artery bypass grafting
Source: Front Surg. 2024 Sep 3;11:1395518. doi: 10.3389/fsurg.2024.1395518 (PMC11405377; doi:10.3389/fsurg.2024.1395518)
Supplement: Supplementary file 1 [file Table1.docx]

| **Table S1 Univariate and multivariate logistic regression analyses of risk factors for perioperative IABP application.** | | | | | | | | |
| --- | --- | --- | --- | --- | --- | --- | --- | --- |
|  | Univariate |  |  |  | Multivariate |  |  |  |
|  | OR | p value | 95% CI |  | OR | p value | 95% CI |  |
| **The overall population** |  |  |  |  |  |  |  |  |
| Age | 1.002 | 0.845 | 0.985 | 1.019 | 0.997 | 0.829 | 0.971 | 1.024 |
| Male | 1.327 | 0.130 | 0.920 | 1.916 | 1.208 | 0.376 | 0.795 | 1.834 |
| BMI | 0.951 | 0.042 | 0.906 | 0.998 | 0.973 | 0.297 | 0.924 | 1.025 |
| HTN history | 0.753 | 0.061 | 0.559 | 1.013 | 0.912 | 0.574 | 0.662 | 1.257 |
| MI history | 1.879 | <0.001 | 1.396 | 2.530 | 1.168 | 0.464 | 0.771 | 1.772 |
| AF history | 1.887 | 0.084 | 0.919 | 3.874 | 1.985 | 0.075 | 0.934 | 4.222 |
| Preoperative SBP | 0.990 | 0.028 | 0.981 | 0.999 | 0.993 | 0.126 | 0.983 | 1.002 |
| Intraoperative EBL≥1000ml | 1.647 | 0.001 | 1.215 | 2.231 | 1.610 | 0.003 | 1.174 | 2.206 |
| LVEF<50% | 4.547 | <0.001 | 3.304 | 6.257 | 3.442 | <0.001 | 2.360 | 5.020 |
| UA | 1.002 | 0.036 | 1.000 | 1.003 | 1.001 | 0.398 | 0.999 | 1.003 |
| Sd-LDL | 1.747 | 0.005 | 1.182 | 2.583 | 2.077 | 0.001 | 1.367 | 3.156 |
| TG | 0.844 | 0.038 | 0.720 | 0.990 | 0.836 | 0.052 | 0.698 | 1.001 |
| EuroSCORE | 1.159 | 0.000 | 1.085 | 1.237 | 1.072 | 0.268 | 0.948 | 1.214 |
| **The elderly subgroup** |  |  |  |  |  |  |  |  |
| Male | 1.810 | 0.030 | 1.058 | 3.098 | 1.591 | 0.097 | 0.919 | 2.755 |
| AF history | 3.045 | 0.007 | 1.347 | 6.885 | 2.900 | 0.013 | 1.252 | 6.717 |
| MI history | 2.374 | 0.000 | 1.504 | 3.748 | 1.907 | 0.010 | 1.170 | 3.107 |
| Intraoperative EBL≥1000ml | 1.772 | 0.014 | 1.121 | 2.803 | 1.802 | 0.014 | 1.125 | 2.886 |
| LVEF<50% | 3.155 | <0.001 | 1.836 | 5.419 | 2.436 | 0.003 | 1.366 | 4.343 |
| **The young subgroup** |  |  |  |  |  |  |  |  |
| BMI | 0.920 | 0.015 | 0.860 | 0.984 | 0.963 | 0.312 | 0.894 | 1.036 |
| HTN history | 0.657 | 0.035 | 0.445 | 0.971 | 0.951 | 0.821 | 0.617 | 1.466 |
| MI history | 1.661 | 0.012 | 1.120 | 2.462 | 1.054 | 0.849 | 0.615 | 1.806 |
| Preoperative SBP | 0.983 | 0.011 | 0.971 | 0.996 | 0.987 | 0.054 | 0.974 | 1.000 |
| Intraoperative EBL≥1000ml | 1.561 | 0.032 | 1.039 | 2.344 | 1.457 | 0.089 | 0.944 | 2.248 |
| LVEF<50% | 5.964 | <0.001 | 3.962 | 8.977 | 5.541 | <0.001 | 3.390 | 9.056 |
| UA | 1.002 | 0.072 | 1.000 | 1.004 | 0.796 | 0.209 | 0.623 | 1.018 |
| Sd-LDL | 1.948 | 0.007 | 1.200 | 3.162 | 2.588 | <0.001 | 1.541 | 4.345 |
| TG | 0.776 | 0.022 | 0.625 | 0.963 | 0.796 | 0.069 | 0.623 | 1.018 |
| HDL-C | 2.017 | 0.074 | 0.934 | 4.357 | 2.017 | 0.111 | 0.852 | 4.777 |
| EuroSCORE | 1.239 | <0.001 | 1.112 | 1.380 | 1.007 | 0.923 | 0.880 | 1.151 |
| **Male subgroup** |  |  |  |  |  |  |  |  |
| BMI | 0.931 | 0.015 | 0.879 | 0.986 | 0.955 | 0.134 | 0.899 | 1.014 |
| HTN history | 0.729 | 0.059 | 0.525 | 1.012 | 0.837 | 0.324 | 0.587 | 1.193 |
| AF history | 2.114 | 0.058 | 0.975 | 4.585 | 2.436 | 0.032 | 1.077 | 5.511 |
| MI history | 1.944 | <0.001 | 1.399 | 2.701 | 1.231 | 0.324 | 0.815 | 1.859 |
| PCI history | 1.455 | 0.089 | 0.945 | 2.242 | 1.336 | 0.212 | 0.847 | 2.107 |
| Preoperative SBP | 0.991 | 0.073 | 0.981 | 1.001 | 0.995 | 0.329 | 0.984 | 1.005 |
| UA | 1.002 | 0.032 | 1.000 | 1.004 | 1.001 | 0.274 | 0.999 | 1.003 |
| Sd-LDL | 1.741 | 0.017 | 1.103 | 2.748 | 1.974 | 0.007 | 1.208 | 3.226 |
| LVEF<50% | 4.692 | <0.001 | 3.305 | 6.662 | 3.589 | <0.001 | 2.411 | 5.343 |
| CAS | 1.378 | 0.066 | 0.979 | 1.940 | 1.343 | 0.142 | 0.906 | 1.992 |
| EuroSCORE | 1.230 | <0.001 | 1.141 | 1.326 | 1.076 | 0.170 | 0.969 | 1.194 |
| Intraoperative EBL≥1000ml | 1.634 | 0.004 | 1.167 | 2.288 | 1.663 | 0.005 | 1.169 | 2.366 |
| **Female subgroup** |  |  |  |  |  |  |  |  |
| LVEF<50% | 3.580 | 0.002 | 1.592 | 8.048 | 3.605 | 0.002 | 1.588 | 8.186 |
| WBC | 0.756 | 0.013 | 0.607 | 0.943 | 0.760 | 0.015 | 0.609 | 0.949 |

**Abbreviations:** BMI: Body mass index; HTN: Hypertension; MI: Myocardial infarction; DM: Diabetes mellitus; AF: Atrial fibrillation; SBP: Systolic blood pressure; EBL: Estimated blood loss; LVEF: Left ventricular ejection fraction; CAS: Carotid artery stenosis; UA: Uric acid; TG: Triglyceride; Sd-LDL: Small dense low density lipoprotein; EuroSCORE: European System for Cardiac Operative Risk Evaluation; PCI: Percutaneous coronary intervention; WBC: White blood cell.

| **Table S2. Interaction analysis of potential risk factors.** | | | | |
| --- | --- | --- | --- | --- |
|  | OR | *p* value | 95% CI |  |
| Overall |  |  |  |  |
| LVEF<50%*sd-LDL | 5.714 | <0.001 | 3.989 | 8.184 |
| The elderly subgroup |  |  |  |  |
| AF history*LVEF<50% | 6.996 | 0.034 | 1.153 | 42.450 |
| MI history*LVEF<50% | 4.359 | <0.001 | 2.342 | 8.113 |
| The young subgroup |  |  |  |  |
| LVEF<50%*sd-LDL | 7.657 | <0.001 | 4.858 | 12.070 |
| The male subgroup |  |  |  |  |
| LVEF<50%*sd-LDL | 5.570 | <0.001 | 3.747 | 8.280 |
